# Biogenesis and functions of circular RNAs and their role in diseases of the female reproductive system

**DOI:** 10.1186/s12958-020-00653-5

**Published:** 2020-11-04

**Authors:** Yalan Ma, Ying Xu, Jingshun Zhang, Lianwen Zheng

**Affiliations:** Reproductive Medical Center, Department of Obstetrics and Gynecology, The Second Hospital of Jilin University, Changchun, Jilin China

**Keywords:** Circular RNAs, Biogenesis, Functions;female reproductive system diseases

## Abstract

A member of the newly discovered RNA family, circular RNA (circRNA) is considered as the intermediate product of by-product splicing or abnormal RNA splicing. With the development of RNA sequencing, circRNA has recently drawn research interest. CircRNA exhibits stability, species conservatism, and tissue cell specificity. It acts as a miRNA sponge in the circRNA-microRNA (miRNA-mRNA axis, which can regulate gene transcription and protein translation. Studies have confirmed that circRNA is ubiquitous in eukaryotic cells, which play an important role in the regulation of human gene expression and participate in the occurrence and development of various human diseases. CircRNA may be closely related to the occurrence and development of female reproductive system diseases. By analyzing the biological functions and mechanism of circRNA, we find that circRNA has certain development prospects as biomarkers of the female reproductive system diseases. The production and degradation of circRNA, biological functions, and their association with the occurrence of diseases of female reproductive system are reviewed in this article.

## Background

Non-coding RNA comprises 95% of the total amount of RNA [1]. Most non-coding RNAs are classified as transcribed super conserved regions and do not participate in protein coding. Non-coding RNA plays a role in gene regulation and promotes the development of various human diseases. In addition to rRNA, tRNA, snRNA and siRNA, new members represented by circular RNA (circRNA) have been identified in the non-coding RNA family. Unlike linear RNA molecules, circRNA is a closed circular molecule without the 5′-3′ polar covalently closed loop structure or the polycyclic adenylate tail [2]. A circRNA is a closed-ring molecule, which renders it more resistant to the RNAse enzyme, compared with other linear RNAs [3]. CircRNAs have drawn considerable interest in RNA field, and this interest is attributed to the new functions found in different cell processes. Specifically, circRNAs may act as a miRNA sponge by blocking the binding of miRNA to target genes, reducing its inhibitory effect on target protein translation [4, 5]. For instance, the testis-specific circRNA Sry has 16 binding sites for miR-138 [6, 7]. This finding changes the understanding of miRNA regulatory mechanism and increases the complexity of competing endogenous RNA (ceRNA) networks. However, the mechanism by which circRNA regulates the network to control its functions has yet to be determined. In addition to miRNA regulation, circRNAs can regulate the intracellular transport of RNA-binding proteins (RBPs) [8, 9]. Notably, some circRNAs may encode functional peptides. Recent studies have shown that circRNAs can be translated in vitro and in vivo [10–12]. Moreover, circRNAs play an important role in various diseases of female reproductive system, such as endometriosis, recurrent miscarriage, repeated implant failure. Research on circRNAs is valuable in the clinical diagnosis and prognosis of these diseases. CircRNAs are expected to be used as biological markers for female reproductive system diseases and as a guide in clinical treatment.

In this article, we summarized the origin, synthesis, and degradation of circRNAs in known mammalian cells. We also studied the function of circRNAs and discussed the expression and mechanism of circRNAs in female reproductive system diseases.

### Discovery and distribution of circRNA

CircRNAs were first identified in 1976 in an electron microscopy-based study of RNA viruses (such as the hepatitis D virus [HDV]) [13]. HDV was first found in some patients infected with the hepatitis B virus in Italy and its structure is covalently linked [14]. Owing to their low abundance and common characteristics, circRNAs were only considered as intermediate products of splicing, by-products, or abnormal RNA splicing events, which failed to attract research attention at the time. CircRNAs became the focus of scientific research with the publication of an article by Hansen TB et al. in the journal *Nature* in 2013. Subsequent research results revealed the considerable potential of circRNAs in biological development [6]. They were found to play a special regulatory role. Studies also demonstrated the presence of thousands of circRNAs in plant cells and their function as negative regulators of their parental genes. According to RNA-seq data, nearly 100,000 circRNAs are expressed in humans [15],w,hich may be related to the alternative splicing of RNA transcripts [16–19]. CircRNAs may come from exonic circRNAs (ecRNAs) [20], intronic circRNAs (ciRNAs) [21], or both exon and intron circRNAs (EIciRNAs) [22]. However, most circRNAs come from the exon gene encoded by proteins and undergo reverse splicing [23]. Among them, There are 5′ end splicing donors connecting upstream 3′ splicing receptors [19, 24]. Unlike most circRNAs, which exist in the cytoplasm, EIciRNAs are mainly located in the nucleus, and this may be related to their regulatory role in the transcription of parental genes [1, 15, 25].

### Biogenesis of circRNAs

**Fig. 1:**
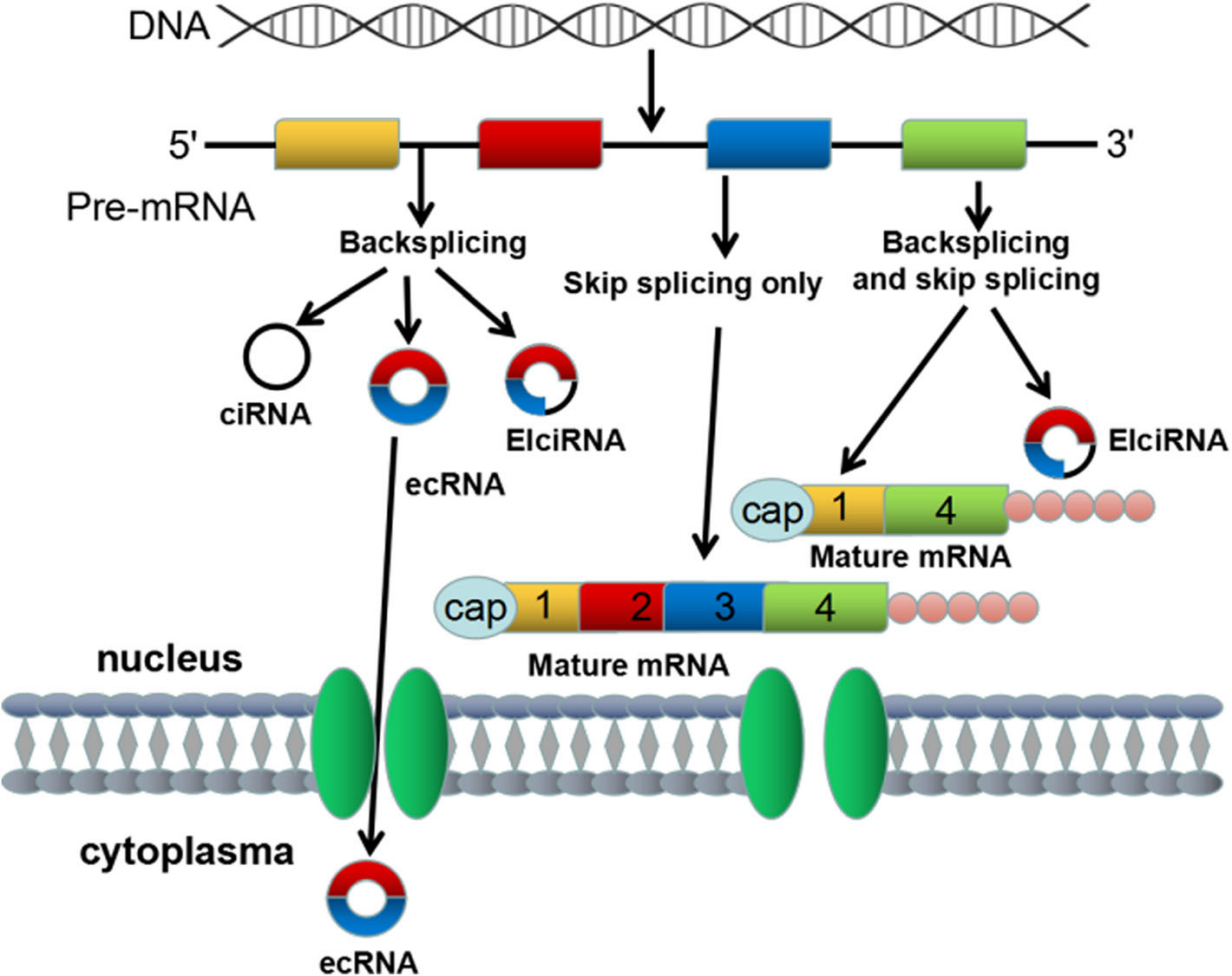
The exons are represented by rectangles of different colors, whereas the introns are represented by black lines. CircRNA may be synthesized in three ways—direct post-splicing, exon jumping, and combined direct post-splicing and exon jumping. The pathway of mature migration to the cytoplasm remains unclear. CircRNA is assumed to pass through the nuclear pore complex across the nuclear membrane

Accumulating evidence shows that circRNA is generated during mRNA processing before splicing [26]. The exons are then reconnected to one another. Owing to the absence of terminal structures such as the 3′ poly (A) tail and the 5′ cap, circRNAs are resistant to endonucleases and thus are more stable than linear mRNAs [27]. Lacking free ends, circRNAs exhibit resistance to endonucleases and are more stable than linear nucleases. CircRNAs are synthesized by direct post-splicing, exon skipping, and the combination of direct post-splicing and exon skipping to participate in the synthesis of ciRNAs, ecRNAs, and EIciRNAs (Fig. 1) [5]. To explain the biogenesis of ecRNAs, two mechanisms have been proposed-exon skipping and direct post-splicing [1, 11, 28]. During exon skipping, downstream exons rotate and skip over one or more exons to connect upstream exons, resulting in a functional mRNA that skips exons. Skipping exons create a lasso precursor with exons and introns, forming circRNAs after the introns are removed [1, 29, 30]. Direct reverse splicing first produces alternative splicing RNAs and lasso intermediates. Introns in the lasso are then removed [15, 31–34]. Recent evidence suggests that direct reverse splicing, rather than exon skipping, is a major mechanism for regulating the formation of ecRNAs [24]. EcRNAs must migrate to the cytoplasm after its biogenesis to exert its regulatory effect. However, the mechanism regulating the migration of mature ecRNAs to the cytoplasm has yet to be determined. The ecRNA output may be regulated by a mechanism similar to the regulation of linear RNA migration [35]. Recent studies show that some types of enzymes are effective. RBPs comprise a group of enzymes involved in the generation of circRNAs in some cases and regulate activation or inhibition [36]. Ashwal–Fluss et al. found that RBPs can monitor the level of a muscleblind-like protein (MBL) in the fly brain. When the muscleblind-like protein level is considerably high, RBPs bind to the former mRNA. This binding prevents linear RNA transformation, halting circRNA translation. MBLprotein does not produce [37]. Adenosine deaminase acting on RNA (ADAR) is another enzyme that participates in the biological processes of circRNA. ADAR can regulate the level of RBPs. Studies have shown that ADAR reduces circRNA formation by weakening and editing RNA double strands [38, 39]. Briefly stated, evidence of evolutionary changes shows that whether or not the complementary sequence is repeated, exon skipping leads to circRNA. This process similarly involves RBPs. Accumulating studies have reported on their contribution to the occurrence and development of diseases, as well as their potential as new clinical diagnosis and prognostic markers [2, 19, 40–42].

### Transport and degradation of circRNAs

**Fig. 2:**
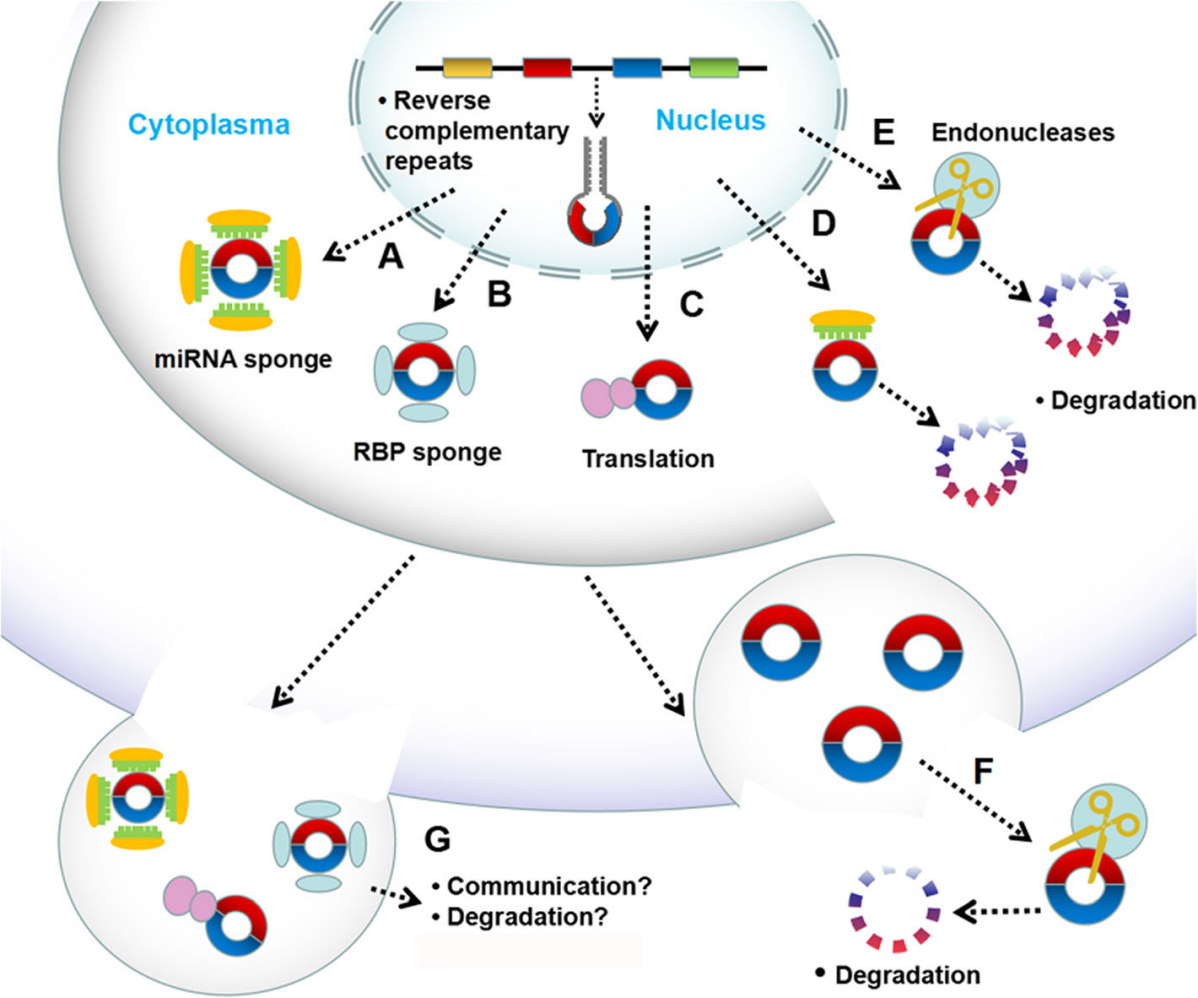
circRNA can be generated either with the help of reverse complementary and exported from the nucleus **a**: Argonaute proteins loaded with miRNAs as sponge **b**: In the cytoplasm,the circRNA might be bound with RNA-binding proteins **c**: translating into peptide in the ribosomes **d**: or direct degradation **e**: causing degradation of the circRNA with the help of endonucleases **f**: the enclosure of circRNAs in vesicle that would be released into the extracellular space would remove circRNAs with the help of endonucleases from the cytoplasm **g**: the circRNAs may reach other cells or tissues and therefore act as messenger molecules or direct degradation

The degradation pathway of circRNAs remains inconclusive. A recent study found that in mammalian cells, circRNAs can be cleared by the release of extracellular vesicles or microbubbles [30]. However, the specific degradation mechanism of circRNAs has yet to be elucidated. As shown in Fig. 2, circRNAs can be produced under a reverse complementary repeat sequence and exported from the nucleus to the cytoplasm. CircRNAs can be combined with RBP, as miRNA sponges or directly degraded in the ribosome, or aided by endonucleases. The circRNA factor complex may diffuse into the cytoplasm, and the circRNAs in the vesicle is released into the extracellular space, removing circRNAs from the cytoplasm. However, protected by vesicles, circRNAs can reach other cells or tissues, acting as messenger molecules, or are directly degraded [43]. CircRNAs have no free ends and thus have no typical RNA decay pathways. Studies on the mechanism and rate of circRNA degradation in vivo are rarely reported. CircRNA degradation can theoretically be initiated by endonucleases [44, 45]. Park et al. showed that the RNA modification N6-methylation of adenosine (M6A) could recruit endonucleases promoting circRNA degradation [46]. Another study also observed circRNA degradation in HeLa cells infected with the encephalomyocarditis virus [47]. Both treatments led to endonuclease activation and circRNA degradation. However, apart from artificial snRNA/siRNA-based systems [1, 10, 12, 48], the only example thus far is the degradation of miR-671 by CDR1as, a circRNA transcribed from the antisense strand of cerebellar degeneration-related protein 1 (CDR1as) [49]. Notably, miRNA binding sites in circRNAs are almost completely proportional to miRNAs. The number of CDR1as is directly regulated by miR-671 via Argonaute2-mediated (AGO2) degradation. CDR1as, miR-671, and their binding sites were highly conserved [6]. The deletion of the site caused a significant increase in the CDR1as level, which may be regulated by miR-7, depending on miR-671 [50]. In autoimmune diseases, the protein kinase R (PKR) phosphorylation level in peripheral blood mononuclear cells is elevated, and circRNAs decrease. In addition to degradation, the elimination of circRNAs from cells can occur by exocytosis. Several studies have detected circRNAs in exosomes [2, 51], and PKR in the extracellular matrix [52]. However, the involvement of circRNA secretion in reducing its intracellular level has yet to be clarified (Fig. 2). Moreover, circRNA secretion may form a communication mechanism (Fig. 2). Therefore, circRNA degradation and extracellular transport should be the focus of future research.

### Biological functions of circRNA

#### Interacting with proteins

**Fig. 3:**
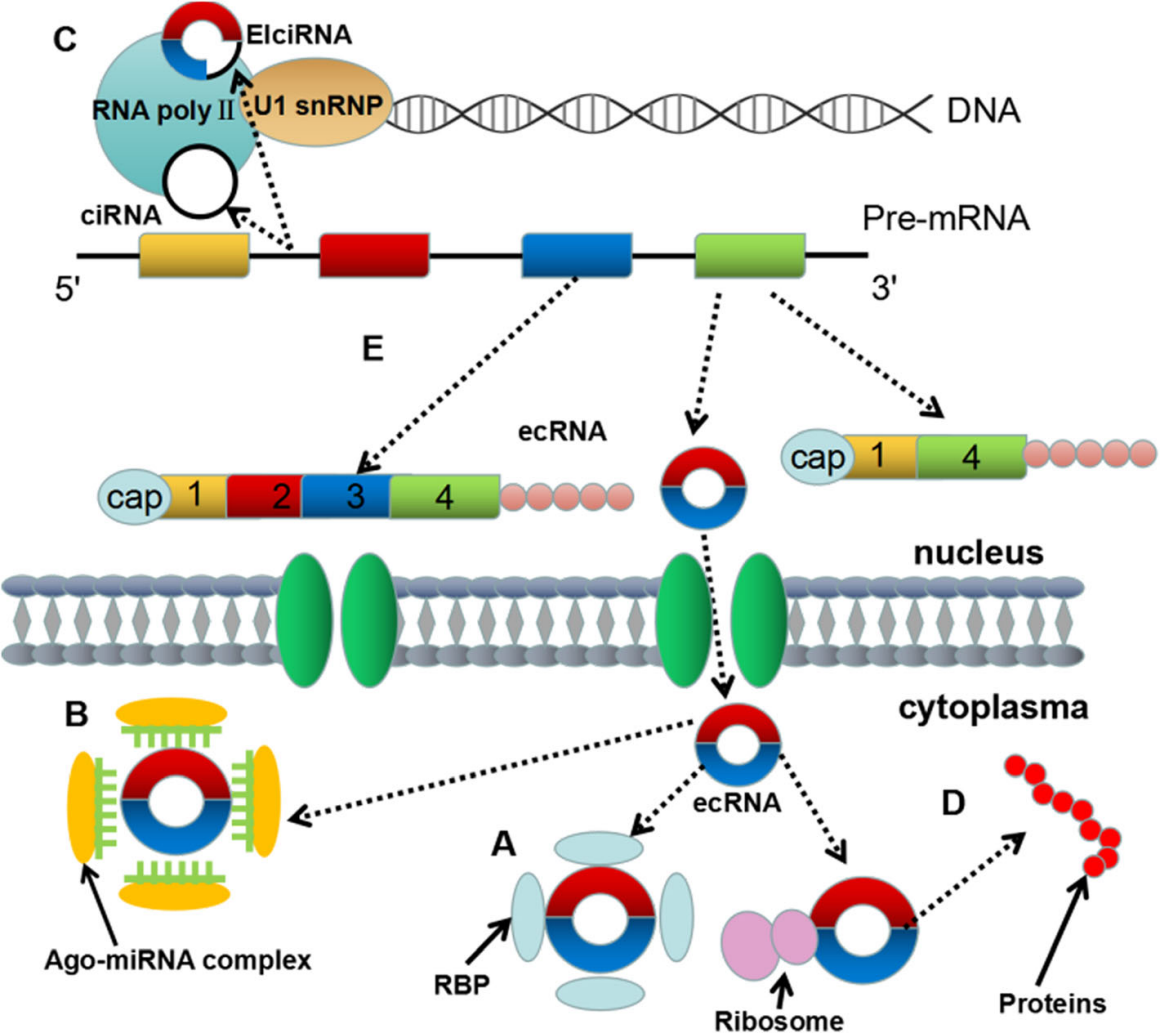
In this figure, exons are represented by rectangles in different colors, and introns are represented by black lines. Five biological functions of circRNAs are proposed: **a** *As an RNA binding protein (RBP) sponge*. To regulate the function of RBPs, some circRNAs can interact with RBPs, such as Argonaute, polymerase II, myosin, and so on. **b** *As a miRNA sponge.* Some circRNA and miRNA binding sites are conserved. By combining with miRNAs competitively, these circRNAs can block binding between the target and the miRNAs. miRNA inhibition of target protein translation. **c** *Regulation of parental gene transcription.* The biogenesis of circRNAs is generally associated with the transcription of its parental genes. Therefore, circRNA can competitively affect the biogenesis and processing of mRNA. **d** *Rolling circle translation*. Some circRNAs contain internal ribosome entry sites, which can be combined with ribosomes. These circRNAs can encode proteins. **e** *To affect alternative splicing*. CircRNA biogenesis can compete with previous mRNA splicing, resulting in reduced linear mRNA [98]

RBPs participate in cell processes (proliferation, differentiation, transport, apoptosis, senescence, oxidative posttranscriptional regulation (alternative splicing and transport) [53]. Stable RNA-protein complexes are formed by circRNA phagocytosis of the Argonaute protein family, RNA fibrillation, MBL protein, RNA polymerase II (POL II), and eukaryotic initiation factors, among others [54]. Some circRNAs work synergistically with RNA. For instance, the binding of cerebellar degeneration protein 1 to cyclin-dependent protein kinase 2 binds circFoxo3 to stop the cell cycle in the G1/S phase. Some circRNAs work synergistically with RNA. For instance, the binding of circFoxo3 to cyclin-dependent protein kinase 2 (CDK2) binds circFoxo3 to stop the cell cycle in the G1/S phase [55]. Some circRNAs, such as circRNA cerebellar degeneration protein 1 (CDR1), can change the stability of mRNAs. CDR1 is used to construct stable double-stranded nucleotide molecules [49]. CircRNAs with mRNA also play a competitive role in regulating the function of RBP (Fig. 3a). HuR is a widely studied RNA-binding proteins (RBPs) that can regulate protein expression patterns by associting with a variety of RNAs. The combination of circPABPN1 and HuR inhibits PABPN1 translation [56]. Moreover, circRNA can bind not only single RBP but also more scaffolds that may act as large proteincomplexes [57]. CircFoxo3 combines with CDK2 and activated kinase (or p21) to form a three-component complex, which inhibits CDK2 function [55].

### As miRNA sponges

The interaction of circRNAs (mainly the Sry circRNA and CIRS-7) as miRNA sponges with miRNAs has increasingly attracted research interest. As an endogenous RNA [58], circRNA uses its miRNA response element (MRE) to mediate the binding between itself and the target miRNA, negatively regulating the expression and biological activity of miRNA and consequently, the expression of target genes [6, 59]. MiRNAs can be combined with 3′ untranslated regions matching the seed area with ceRNAs, suggesting that each circRNA has multiple miRNA target sites. By competing with miRNAs, circRNAs indirectly regulates mRNA translation (Fig. 3b). CDR1as/CIRS-7, a circRNA transcribed from the antisense strand of CDR1, widely exists in human or mouse brains. CIRS-7 contains 74 miR-7 binding sites, which can act as miR-7 sponges and inhibit its biological function [49]. The expression of miR-7 is increased. Capel et al. found that the sex-determining gene Sry on the Y chromosome could be transcribed. Hansen et al. verified that Sry circRNA can act as a miRNA sponge and contains 16 sites that can bind to miR-138 [60–63]. These findings change the understanding of the mechanism of miRNA regulatory network and increase the complexity of the competitive endogenous RNA (ceRNA) network [8]. These findings change the understanding of the mechanism of the miRNA regulatory network and enhance the complexity of the ceRNA network [64].

### Regulation of parental gene transcription

Almost all circRNAs distributed in the cytoplasm are produced by exons [2, 32]. CiRNAs and EIciRNAs are mainly located in the nucleus, most likely functioning at the transcription level [21, 32]. EIciRNAs interact with U1 small ribonucleoproteins (U1 snRNPs) and POL II via the U1 snRNA binding site and perform similar cis-regulatory functions [23]. CiRNAs are mainly located in the nucleus and almost have no miRNA binding functions; notably, ciRNA knockout can hinder the transcription of the corresponding gene. Ci-ankrd52 is abundantly enriched in the transcription initiation region of related genes and promotes Pol II function. Ci-ankrd52 has been preliminary proved to promote the transcription of corresponding genes [65]. It also shows that circRNAs can not only interact with the Pol II complex in the nucleus by regulating the cis or trans transcription of their parent genes; circRNAs also positively regulate Pol II transcription (Fig. 3c) [66]. The ciRNA and EIciRNA located in the nucleus regulate gene transcription through various mechanisms. Studies have shown that in HEK293 and Hela cell nuclei cultured in vitro, EIciRNA (circ-ETF3 and circ-PAIP2) can bind to U1 snRNP and cis-regulate the parental gene. EIciRNAs, aided by U1snRNA [66], promote the transcription of their parental genes in cis. U1 snRNA can mainly play a role via the specific RNA-RNA interaction between U1 snRNA and EIciRNAs. Simultaneously, EIciRNA downregulation can reduce its parental mRNA expression, further suggesting that EIciRNAs exert a positive effect on the transcription of its parental mRNA. In addition, circ-sirt7 also acts as a cis-acting element to enhance the activity of Pol II, promoting the transcription of its parent gene [67].

### Rolling circle translation

The majority of circRNAs produced by reverse splicing are mainly located in the cytoplasm [1, 15], and whether they are translatable is a concern. Linear mRNA translation usually requires a 5′ terminal 7-methyl guanosine cap structure and a 3′ poly-A tail. CircRNAs has neither a cap nor a poly-A tail; thus, its translation is suggested to occur independently of the cap. One method to achieve circRNA translation is to promote the direct binding of initiation factors or ribosomes to translatable circRNAs via the sequence of internal ribosome entry sites (IRESs). In vivo and in vitro experiments have demonstrated that the 40S subunit of eukaryotic ribosomes can be combined with circRNAs containing ribosome entry sites [11, 68]. Similarly, in *Escherichia coli*, transfection of circRNAs carrying the green fluorescent protein open reading frame can activate the expression of the green fluorescent protein. Only viral circRNAs have thus far been found to encode proteins in eukaryotic cells [68]. The HBV satellite virus HDV can be translated by co-transfection with HBV in host cells [69]. The translation of virus circRNAs may be related to specific viral media. Although their ability to translate proteins remains undetermined, some eukaryotic circRNAs have demonstrated their potential, as verified by a transcript of circRNA synthesis (Fig. 3d). M6A can promote the effective initiation of circRNA protein translation in human cells [70]. Legnini et al. also found that circ-ZNF609 in the mouse myoblast nucleus binds to the ribosome via its upstream ribosome entry sites and participates in protein translation [10]. Moreover, during the artificial synthesis of circRNAs lacking ribosome entry sites, multiple FLAG coding sequences can be inserted to allow circRNAs to translate proteins by rolling circle amplification. In addition, after treatment with puromycin, the circZNF609 in myoblasts changes to a lighter ribosome pattern similar to that of the corresponding ZNF609, indicating active translation [10].

### Alternative splicing

A large portion of the circRNAs we have identified is thus far derived from exons. Therefore, the formation of this circRNA may affect the selective splicing of related precursor mRNA (pre-mRNA), which may lead to changes in gene expression (Fig. 3e). Although reverse splicing is not as beneficial as normal splicing, the use of 5′ and 3′ in circRNA biogenesis can compete with pre-mRNA fusion, resulting in reduced levels of linear mRNAs containing exons [71] In general, the greater the number of cycles an exon undergoes, the less it appears in the processed mRNA [30]. However, not all scheduled exons can produce circRNAs, suggesting that additional regulatory factors can influence bypassing cyclone cycling or linear isomers. Under endogenous conditions, the degree of correlation between exon circulation and exon sponsorship should be determined, and whether such events would lead to observed biological effects should be ascertained. By removing specific exons from pre-mRNA, the composition of mRNA after processing is changed [72]. No natural eukaryotic circRNA has the function of translating into protein; however, as previously mentioned, the more cyclized an exon, the less it appears in the processed mRNA. Thus, we speculate that overexpression or deletion of some gene splicing-related proteins may also regulate mRNA formation by controlling the number of circRNAs. This process further regulates gene expression. This theory can provide an insight into the study of the pathogenesis of certain diseases.

### Expression of circRNAs in diseases of the female reproductive system

#### Expression of circRNAs in pre-implantation embryos

CircRNA expression in preimplantation embryos has thus far been reported in mice and humans. A total of 2891 circRNAs and 913 new transcripts have been found in mouse preimplantation embryos. Most circRNAs are distinct from the preimplantation stage, and a large proportion of has shown a dynamic expression pattern during this developmental process [73]. CircRNAs are relatively abundant in preimplantation embryos, and thousands of copies of circRNAs are present in each embryo. These circRNAs may participate in the chromosomal organization, cell cycle adjustment, and DNA repair of early-phase mouse embryos. These circRNAs exhibit distinct characteristics, suggesting their peculiar patterns [25]. The abundance of circRNAs in mouse oocytes and preimplantation embryos fluctuate, which may be attributed to their special roles in different developmental stages. Similar methods were used to detect 10,032 circRNAs from 2974 host genes in human preimplantation embryos on the basis of differentially expressed genes [38]. A total of 1554 maternal genes and 851 zygotic genes were identified as host genes. The occurrence of circRNAs between humans and mice is usually conserved. Most biological functions of circRNAs detected in preimplantation embryos remain unclear. These circRNAs may regulate miRNA gene expression as circRNA sponges during embryo development. Dang et al. also found a large number of circRNAs transcribed from maternal genes, most of which existed before fertilization. They may be resistant to maternal mRNA degradation. Compared with those in mice, circRNAs in humans have been proved to be both conserved and more complex, indicating that they are conserved and specific in human preimplantation development [74].

#### Expression in granule cells

Granulosa cells (GCs) play an important role in oocyte maturation and early embryo development [75, 76]. Some studies show that the gene expression pattern in GCs can provide new potential diagnostic and therapeutic targets for polycystic ovary syndrome (PCOS). Qi et al. examined the expression pattern of circRNAs in the GCs of patients with PCOS was screened by circRNA microarray analysis, and the GC expression levels in 20 PCOS patients and 20 non-PCOS patients were determined [77]. Moreover, of the 1032 that were differentially expressed in cumulus cells (GC), 311 were upregulated, and 721 were downregulated. Four abnormally expressed circRNAs showed statistical significance. In addition, 4 circRNAs showed statistically significant results: hsa_circ_0083952, hsa_circ_0082709, hsa_circ_0002425, and hsa_circ_0015168, which were confirmed by quantitative reverse transcription–polymerase chain reaction (RT-PCR). This finding suggested that they were closely related to PCOS. As target miRNA sponges, circRNAs may play a role in the circRNA-miRNA gene network, as well as bind to miRNA in the mRNA-competitive cytoplasm, thereby interfering with gene regulation. Gene ontology (GO) enrichment data show that the aforementioned abnormal expression of circRNA may be involved in important biological functions. In Kyoto Encyclopedia of Genes and Genomes (KEGG) pathway analysis, the metabolic pathway is the most important route of enrichment. PCOS is associated with an increased risk of metabolic abnormalities, including insulin resistance, hyperinsulinemia, type 2 diabetes, and dyslipidemia;the specific mechanism requires further study [78]. Moreover, the nuclear coding genes involved in mitochondrial oxidative metabolism are confirmed to be downregulated in the skeletal muscles of women with PCOS [79], Thus, the association of these abnormally expressed circRNAs with mitochondrial dysfunction has to be determined. Wang et al. for PCOS in future work. A total of 16,771 candidate circRNAs in follicular fluid exudates of patients were analyzed by high-throughput sequencing. Compared with normal female follicular fluid, 167 upregulated circRNAs and 245 downregulated circRNAs were detected in the follicular fluid of PCOS patients. Functional analysis showed that the pathways related to bacterial infection, slow inflammation, and oxidative stress could be regulated by these differentially expressed circRNA targets. The sequencing results were further verified by constructing the circRNA-miRNA network. Moreover, circRNA_103827 predicted the pregnancy outcome after assisted reproductive technology, indicating its potential as a new biomarker for predicting the outcome of in vitro fertilization embryo transfer [80].

### CircRNAs and diseases related to the female reproductive system

#### CircRNAs and endometriosis

Endometriosis (EMT) is a challenging disease characterized by dysmenorrhea and infertility. However, its etiology has yet to be clarified, and no effective markers or treatments exist. It affects the reproductive outcomes for women of childbearing age and exhibits the malignant characteristics of adhesion, invasion, and angiogenesis [81, 82]. Xu et al. found that endometriosis is a commonly occurring disease. In their study, 88 differentially expressed circRNA were found in the ectopic endometrium, 11 of which were upregulated, and 77 were downregulated. These findings revealed the role of the EMT circRNA expression pattern and the circRNA-miRNA-mRNA network in the ovary [83]. The study suggested that circRNA played a key role in the pathogenesis of ovarian EMT and verified that circ_0004712 and circ_0002918 were upregulated. It could become an organism for diagnosing ovarian endometriosis. Marker gene chip analysis also revealed that circ_0004712,circ_0002198, circ_000891 circ_0017248,and circ_0003570 were differentially expressed in ectopic endometrium and eutopic endometrium. These differentially expressed circRNAs regulate various genes and miRNAs, such as TP53, consequently regulating the various biological functions of the endometrium. In these circRNAs, circ_0004712 and circ_0002198 induced apoptosis and cell cycle arrest, as well as inhibited the proliferation, angiogenesis, and contraction of ovarian endometrial stromal cells. Circ_0004712 can reduce oxidative stress, as well as inhibit apoptosis and autophagy, by upregulating its own target gene VDR. A previous study also indicated that VDR was regulated in the endometrium. Circ_0008951 affected the endocrine function and regulated the thyroid hormone receptor pathway. This pathway plays an important role in ovarian epithelial endoderm transplantation. Moreover, circ_0008951 targets miR-29c and prompts a reduction in the progesterone activity in the endometrium. In endometrial carcinoma, circ_0017248 inhibits cell proliferation and invasion by targeting miR-145. Circ_0003570 originates from the MBOAT1 gene and positively regulates PPP1R9A and SOX4. This interaction between circRNA and miRNA is not one-to-one. A circular RNA can target multiple miRNAs, and one miRNA can be regulated by multiple cyclic RNAs. For instance, miR-503 can induce apoptosis, block the cell cycle, as well as inhibit cell proliferation and angiogenesis by using circ_0004712 and circ_0002198 together. Zhang et al. selected 20 cases of ovarian endometriosis and 4 non-endometriosis cases [84]. By using microarray analysis, 2237 circRNAs were detected in the endometriosis group; differences were obvious between the 3 groups. Eight RT-PCR methods were found to participate in epithelial-mesenchymal transition. The results indicated that circ_103470 and circ_101102 showed identical gene chip results; the 2 circRNAs were downregulated. Epithelial-mesenchymal transition may be regulated in endometriosis through miR-1415p, which can potentially be a therapeutic target for circ_101102. Circ_101102 is reported to inhibit autophagy in mammalian cells [85]. It induces autophagy in normal endometrial tissues throughout the menstrual cycle. Abnormal autophagy can occur in eutopic endometrium and ectopic endometrium in endometriosis. CircRNA regulation is suggested to be involved in the pathogenesis of endometriosis by inducing autophagy. The circ_103237 target miR-34 inhibited cell proliferation, migration, and cell cycle maintenance in the early secretory phase in endometriosis (Table 1).

**Table 1:** Expression and clinical application of cirRNAs related to diseases in the reproductive system

Reproduction diseases	Circular RNA	Regulation of the expression in reproductive diseases	miRNA	Biology function	References
Ovarian Endometriosis	circ_0004712	Up	miR-503	Induce apoptosis and cell cycle arrest, as well as inhibits ovarian endometrial stromal cell proliferation, angiogenesis, and contraction.	[84]
	circ_0002918	Up	miR-503		
	circ_0008951	Up	miR-29c	Inhibition of cell proliferation and invasion.	[82]
Repeated Implantation Failure	hsa_circRNA_103716、	Up	hsa-miR-574-5p	miR-574-5p overexpression inhibits cervical cancer growth and metastasis.	[86]
	hsa_circRNA_ 070616	Up	hsa-miR-574-5p	miR-574-5p overexpression inhibits cervical cancer growth and metastasis.	[86]
	circRNA-9119	Up	miR-26a	miR-26a downregulates the expression of Ptgs2 in endometrial epithelial cells (EECS) of dairy gpredicted targets.	[87]
Recurrent spontaneous abortion (RSA)	hsa_circRNA_104792	Down	miR-133a	Used as RSA biomarkers.	[88]

### Circular RNAs and repeated implantation failure

Approximately 15% of women in their reproductive years worldwide are affected by infertility [87, 89]. Numerous women have good embryos, but some have repeated implantation failures, which may be related to endometrium-related factors [88]. Therefore, it is necessary to pay attention to the endometrial receptivity molecule in patients with recurrent implantation failure. Qiao et al. found that circRNAs are involved in oocyte development and embryo implantation [74]. Liu et al. detected multiple differentially expressed circRNAs in repeated implantation failure specimens, with hsa_circ_103,716 and hsa_circ_070616 as overexpressed circRNAs [86]. Two circRNAs could be used hsa_miR-574-5p sponges by reducing the expression of MACC-1. Invasion and metastasis of non-small cell lung cancer and colorectal cancer are inhibited. In gynecological diseases, miR-574-5p overexpression inhibits the growth and metastasis of cervical cancer. Notably, Zhang et al. reported that hsa_miR-574-5p plays an important role in endometrial hyperplasia [90]. It can be used as a biomarker for overexposure to estrogen. Several studies reported that estrogen supplementation could improve endometrial thickness in patients with recurrent implantation failure [91]. Endometrial receptivity is crucial for implantation [92]. The acquisition of endometrial receptivity is a complex process [93, 94]. In the study by Zhang et al., circRNA_9119 as a microRNA sponge reduced the miR-26a level. MiR-26a reduced prostaglandin-endoperoxide synthase 2 (PTGS2) expression in the endometrial epithelial cells (EECs) of dairy goats in vitro by using the predicted target. In this manner, circRNA-9119 played a ceRNA role in vitro in goat milk EECS and isolated miR-26a, protecting the prostaglandin-endoperoxide synthase 2 transcript from miR-26a-mediated inhibition. Ptgs2 is also involved in the regulation of several protein markers of endometrial receptivity in dairy goat culture. Therefore, the circRNA_9119/miR-26a/Ptgs2 pathway is found in the endometrium. Regulating circRNA_9119/miR-26a/Ptgs2 expression in EECS can be a potential target for controlling the occurrence of repeated implantation failures (Table 1) [95].

### Circular RNAs and recurrent spontaneous abortion (RSA)

RSA is a commonly occurring disease in women worldwide. Qian et al. chose 35 pairs of patients with RSA and healthy controls. The abnormal expression of circRNA in 3 villi tissues was analyzed using a circRNA chip. A total of 594 abnormally expressed circRNAs were detected, 335 of which were upregulated. The downregulation of 259 circRNAs in the microarray showed numerous circRNA miR-133a binding sites [96]. Wang et al. found that the miR-133a expression in patients with RSA was significantly higher than that in healthy pregnant women. MiRNA-133a might interact with HLA-G 3’UTR to downregulate HLA-G expression, prompting the occurrence of RSA [97, 98]. CircRNA_104792 is a downregulated circRNA, with a miR-133a binding. The low circRNA_104792 expression weakens the inhibitory effect of miR-133a on its target genes and participates in the occurrence of RSA. With the interaction between miRNAs and circRNAs, these circRNAs can bind to the corresponding miRNAs and weaken their inhibitory effect on target genes via their miRNA sponges, affecting the pathogenesis of RSA. ROC analysis confirms the presence of 8 circRNAs, including circRNA_104948, circRNA_104792 are downregulated circRNAs, with a miR-133a binding with hsa-circRNA. The low circRNA_104792 expression weakens the hsa_circRNA_104547, and hsa_circRNA_101319, which exhibit good sensitivity and specificity and can be used as biomarkers for RSA (Table 1).

## Conclusion

As a member of the newly discovered RNA family, CircRNA has shown its distinct advantages and has drawn research interest in RNA research because of its stability, high specificity, and conservative sequence. However, research on circRNA has yet to be developed. Various types of circRNA have been identified; however, few circRNA biological functions have been determined. Most mechanisms are still in the research stage. The biological functions of RNA remain largely unknown. The potential functions of circRNA-as a miRNA sponge, as RNA binding protein sponge, and rolling circle translation-have added a regulatory network to cell function. The coding ability and stability of circRNAs, which can be used in biotechnology requiring peptide production with the steady development of RNA technology, the circRNA field is anticipated to considerably develop in the next few years. The location, transportation, and degradation in the living cells of circRNAs, as well as the complete circRNA interactive body are expected to be elucidated. The association of circRNA with ovarian endometriosis, repeated reproductive failure and recurrent abortion, has been revealed gradually. However, the whole remains in the initial stages of research. Only several circRNA families participate in the development of diseases related to the female reproductive system. More in-depth studies need to be conducted to confirm the role of circRNA in the pathogenesis of female reproductive diseases and present innovations in the diagnosis and treatment of diseases related to the female reproductive system. CircRNAs can be secreted into the extracellular environment and detected in blood, urine, tissues, and secretions by non-invasive means. This finding indicates that a specific circRNA may be used as a biomarker and a disease prognostic indicator. Researchers can present a more detailed and in-depth study of the known function of circRNAs, as well as provide new evidence and direction for clinical diagnosis, treatment, and prognosis prediction by discovering the molecular mechanisms of circRNAs.

## Data Availability

This review was based on published data.

## Abbreviations

ADAR: Adenosine deaminase acting on RNA
AGO2: Argonaute2
CDR1: Cerebellar degeneration protein 1
CDR1as: A circRNA transcribed from the antisense strand of cerebellar degeneration-related protein 1
CDK2: Cyclin-dependent protein kinase 2
ciRS-7: circRNA for miRNA-7
circRNA: circular RNA
ceRNA: competing endogenous RNA
ecRNA: Exonic circRNA
EECs: Endometrial epithelial cells
EIciRNAs: Both exon and intron circRNAs
EMT: Endometriosis
GCs: Granulosa cells
HDV: hepatitis D virus
IRES: Internal ribosome entry site
KEGG: Kyoto Encyclopedia of Genes and Genomes
M6A: Modification N6-methylation of adenosine
MBL: muscleblind-like RNA
MRE: miRNA response element
P21: Activated kinase
PCOS: Polycystic ovary syndrome
PKR: Protein kinase R
POL II: Polymerase II
PTGS2: Prostaglandin-endoperoxide synthase 2
RBPs: RNA-bindingproteins
RSA: Recurrent spontaneous abortion
U1 snRNP: U1 small nuclear ribonucleo protein particle
